# The Boarding out of Pauper Children

**Published:** 1889-09-07

**Authors:** 


### The Boarding Out of Pauper Children.

### I.—THE SYSTEM ITSELF.

*(Continued from page 341.)*

At present the boarding-out system, so far as boards of guardians are concerned, is limited to "orphan and deserted children," and these descriptions are not applied quite in the ordinary sense. In the case of illegitimate children, an orphan is a child whose mother is dead, and a deserted child is one whose mother is transported, or insane, or bedridden, and permanently residing in a workhouse. In the case of legitimate children, an orphan is a child both of whose parents are dead, or one of whose parents is dead, the other being in penal servitude, or insane, or a permanently disabled inmate of a workhouse; and a deserted child is one who is deserted by both parents, or deserted by one parent, the other being dead or under the disabilities mentioned above, or both of whose parents are under these same disabilities.

If the boarding-out system should show results more and more satisfactory, there can be little doubt that it will be largely extended in the near future. And it is to secure these better results, and to secure them more certainly and uniformly, that the new regulations have been issued by the Local Government Board. These regulations go not only to the root of the matter, but to all the branches of it as well. With one stroke of the pen all previous provisions are cancelled, so that a definite and clear course may be had for the running of the new plan. Committees of ladies and gentlemen will have written authority from the Local Government Board to enter into arrangements with boards of guardians for the finding and superintending of homes for pauper children. These voluntary workers, through their secretary, will keep the Local Government Board fully informed of their proceedings, and at least twice a year formal reports from them will be expected at headquarters. The authority of the guardians, however, is well kept in view, both in this and all other details of the scheme. The guardians, if they see fit, can remove any child from any home on giving the committee one week's notice of their intention to do so, and foster-parents, in this important matter of giving up children on demand, will be bound under their contract to respect the authority, whether of the guardians or of the committee.

In arranging with foster-parents for the maintenance of children, the guardians are limited to the payment of four shillings per week. Although this sum is exclusive of expense for clothing, school fees, medical attendance, medicines, extras, etc., many will consider it insufficient for the comfortable maintenance and lodging of a child from two to ten years old. And, as a matter of fact, it has happened in one instance that a suitable home could not be found under 6s. per week for each child, and in this case the committee generously provided the extra money in order to secure really suitable surroundings for the children. Generally, however, and in the homes which are most desirable for the purpose, the 4s. limit is no obstacle. How many agricultural labourers, even in harvest-time, can devote 4s. a week to the lodging and maintenance of each of their own children, and, of course, for the rest of the year, nothing approaching this is possible. It has been truly said that the boarded-out children will be much better provided for than the children of an ordinary agricultural labourer, and as truly added: "Considering how much such children are handicapped by the circumstances of their birth and early rearing, they need not be grudged some slight advantage of this kind."

The committee will use their own judgment, instructed by an intimate knowledge of the neighbourhood, in selecting as foster-parents respectable, honest, and industrious people, apt to keep their engagement to bring up a child boarded out with them as one of their own. The perils of baby-farming are evidently contemplated in certain regulations which will also assist the choice of committees in this part of their duties. No one who has been in receipt of relief for twelve months preceding will be eligible as a foster-parent, and there are very definite restrictions upon the number of children for one home.

"Not more than two children shall be boarded out by the guardians in the same home at the same time, unless all such children are brothers and sisters, and do not exceed four in number; not more than one child shall be boarded out by the guardians in a home in which any child is boarded out by persons other than the guardians, nor shall any child be boarded out in a home in which there is more than one such child; and no child shall be boarded out in a home in which, at the time when the child would first be placed in it, there would be with such child more than five children resident."

The foster-parent, it is likewise provided, shall be of the same religious persuasion as the child, and it will be part of his engagement to see "that the child shall duly attend at church or chapel according to the religious creed to which the child belongs." One penny a week (for each child) is to be allowed to the master of any school attended by these children, in consideration of a special quarterly report to the guardians of the school progress, attendances, etc., of the children. There is also provision made for the regular medical inspection and attendance of the children, and a strict regulation as to the smallest allowable supervision by the committee.

"Every boarded-out child shall be visited not less often than once in every six weeks by a member of the Boarding-out Committee at the home of the foster-parent, and the visitor shall thereupon make a report in writing to the committee stating the apparent bodily condition and the behaviour of such child, and all reasonable complaints made by or concerning the child against or by the foster-parent."

Though we have given but the leading points in the Local Government Board Orders, it will be fully apparent that the utmost care is taken to prevent any abuse, and that we have entered upon a new era of the boarding-out system. We should gladly give some details of the efficiency, kindness, and generosity which have marked the work of the various committees throughout the country; but these details, as well a fuller review of Miss Mason's comprehensive report, must be reserved for another article.

